# The Impact of Full-Length, Trimeric and Globular Adiponectin on Lipolysis in Subcutaneous and Visceral Adipocytes of Obese and Non-Obese Women

**DOI:** 10.1371/journal.pone.0066783

**Published:** 2013-06-21

**Authors:** Zuzana Wedellova, Zuzana Kovacova, Michaela Tencerova, Tomas Vedral, Lenka Rossmeislova, Michaela Siklova-Vitkova, Vladimir Stich, Jan Polak

**Affiliations:** 1 Department of Sport Medicine, Third Faculty of Medicine, Charles University in Prague, Czech Republic; 2 2^nd^ Internal Medicine Department, University Hospital of Kralovske Vinohrady, Prague, Czech Republic; 3 General Surgery Department, University Hospital of Kralovske Vinohrady, Prague, Czech Republic; 4 Franco-Czech Laboratory for Clinical Research on Obesity, Third Faculty of Medicine and INSERM Unite 586, Charles University, Prague, Czech Republic; 5 Division of Pulmonary and Critical Care Medicine, Johns Hopkins University, Baltimore, Maryland, United States of America; University of Colorado Denver, United States of America

## Abstract

Contribution of individual adiponectin isoforms to lipolysis regulation remains unknown. We investigated the impact of full-length, trimeric and globular adiponectin isoforms on spontaneous lipolysis in subcutaneous abdominal (SCAAT) and visceral adipose tissues (VAT) of obese and non-obese subjects. Furthermore, we explored the role of AMPK (5'-AMP-activated protein kinase) in adiponectin-dependent lipolysis regulation and expression of adiponectin receptors type 1 and 2 (AdipoR1 and AdipoR2) in SCAAT and VAT. Primary adipocytes isolated from SCAAT and VAT of obese and non-obese women were incubated with 20 µg/ml of: A) full-length adiponectin (physiological mixture of all adiponectin isoforms), B) trimeric adiponectin isoform or C) globular adiponectin isoform. Glycerol released into media was used as a marker of lipolysis. While full-length adiponectin inhibited lipolysis by 22% in non-obese SCAAT, globular isoform inhibited lipolysis by 27% in obese SCAAT. No effect of either isoform was detected in non-obese VAT, however trimeric isoform inhibited lipolysis by 21% in obese VAT (all p<0.05). Trimeric isoform induced Thr172 p-AMPK in differentiated preadipocytes from a non-obese donor, while globular isoform induced Ser79 p-ACC by 32% (p<0.05) and Ser565 p-HSL by 52% (p = 0.08) in differentiated preadipocytes from an obese donor. AdipoR2 expression was 17% and 37% higher than AdipoR1 in SCAAT of obese and non-obese groups and by 23% higher in VAT of obese subjects (all p<0.05). In conclusion, the anti-lipolytic effect of adiponectin isoforms is modified with obesity: while full-length adiponectin exerts anti-lipolytic action in non-obese SCAAT, globular and trimeric isoforms show anti-lipolytic activity in obese SCAAT and VAT, respectively.

## Introduction

Excessive accumulation of adipose tissue is associated with development of multiple metabolic and cardiovascular abnormalities including insulin resistance and type 2 diabetes [1], [2]. Even though exact pathogenesis of metabolic impairments in obesity is not fully elucidated, elevated plasma levels of free fatty acid (FFA) has been recognized as a possible causal link [3], [4].

Higher plasma FFA levels were repeatedly observed in obese and insulin-resistant subjects compared to lean individuals, probably due to increased delivery of FFA from enlarged adipose tissue [5]–[7]. Several impairments in the control of lipolysis were documented in obese subjects including altered sensitivity to major regulators of lipolysis such as catecholamines, insulin and natriuretic peptides [4], [8]. Moreover, cytokines secreted directly from adipose tissue have been recognized as potent paracrine regulators of lipolysis. Tumor necrosis factor-α and interleukin-6 were shown to stimulate lipolysis *in-vitro* and *in-vivo* [9]–[12]. Recently, our group has shown that adiponectin - a cytokine with marked anti-inflammatory, anti-atherogenic and insulin-sensitizing properties [13], inhibits spontaneous as well as catecholamine-induced lipolysis in non-obese subjects, while this effect was lost in obesity [14].

During production in adipocytes, individual adiponectin molecules (monomers with molecular weight of ∼30 kDa) form distinct polymeric isoforms detectable subsequently in human plasma. Multimeric isoforms composed of 12–18 monomers together with hexameric and trimeric isoforms are abundant in circulation [14]–[16], while a globular fragment produced by proteolytic cleavage of adiponectin is detectable in small quantities [13], [17], [18]. Although the biological function of individual isoforms is not sufficiently understood, it has been reported that adiponectin-induced activation of 5'-AMP-activated protein kinase (AMPK), a central effector in adiponectin signaling cascade, is differentially modulated by individual polymeric isoforms in various tissues [19]–[21]. For example: multimeric isoform, but not globular fragment, effectively decreased blood glucose through inhibition of hepatic glucose output and stimulation of glucose uptake in muscle [22]. Furthermore, multimeric isoform is associated with insulin sensitivity indices and improvements in glucose metabolism induced by thiazolidinedione treatment [22]. In contrast, globular fragment, but not multimeric isoform, enhanced fatty acid oxidation in skeletal muscle [17], [21]–[23].

Multiple metabolic pathways, including lipid and glucose metabolism, are regulated through AMPK. Upon activation, AMPK specifically phosphorylates several downstream enzymes, such as hormone sensitive lipase and acetyl-coenzyme A carboxylase leading to stimulation of lipogenesis and inhibition of lipolysis [14], [24]. Although adiponectin-induced activation of AMPK was documented in human and mouse adipocytes [14], [24], [25], the role of individual adiponectin isoforms remains unknown.

We designed this study to elucidate the role of adiponectin isoforms in paracrine lipolysis regulation. First, we investigated the impact of globular, trimeric and full-length adiponectin on spontaneous lipolysis in adipocytes derived from subcutaneous and visceral adipose tissue of obese and non-obese women. Second, we explored whether individual polymeric isoforms induce AMPK activation in differentiated preadipocytes obtained from obese and non-obese donors. Finally, we investigated distribution of adiponectin receptors in SCAAT and VAT of both groups.

## Methods

### Subjects

Samples of subcutaneous abdominal (SCAAT) and visceral adipose tissue (VAT) were obtained during planned laparoscopic surgery (cholecystectomy, hernioplasty) from obese (SCAAT N = 10, VAT N = 6) and non-obese (SCAAT N = 8, VAT N = 8) women. All subjects were without any chronic medication and free of any disease-related symptoms as assessed during physical examination by a physician.

### Ethics Statement

All participants gave written informed consent before participation in the study and the study was performed in accordance with the Declaration of Helsinki and the Part 46, Title 45, U.S. Code of Federal Regulations. The study was approved by the Ethics Committee of the Third Faculty of Medicine, Charles University (Prague, Czech Republic).

### Adipocyte Isolation

Following biopsy, samples were immediately submerged in sterile saline and transported to the laboratory for subsequent experiments. Isolation of primary adipocytes followed previously described protocol [14]. Briefly, the whole tissue was cut into ∼ 1–2 mm pieces and subsequently digested for 30 min at 37°C under gentle shaking (100 cycles/min) using 1.25 mg/ml collagenase (Collagenase from Clostridium histolyticum, Sigma-Aldrich, Prague, Czech Republic) dissolved in KRBHA buffer (Krebs Ringer Bicarbonate buffer with 10 mM HEPES, 2% fatty acid free bovine serum albumin (Sigma-Aldrich, Prague, Czech Republic) and 6 mM glucose, pH 7.4). After digestion, adipocytes were filtered through a 250 µm silk screen and washed 3 times with KRBHA buffer to eliminate undigested tissue and dead cells.

### *In-vitro* Incubations and Lipolysis Determination

Freshly isolated adipocytes were divided into five aliquots, each containing 95 µl of cell suspension and 80 µl of KRBHA buffer. Cells were left to recover from digestion for one hour and subsequently incubated for 2 hours as follows: A) KRHBA buffer without any pharmacological intervention (control experiment), B) KRHBA buffer +20 µg/ml of full-length adiponectin representing a physiological mixture of multimeric, hexameric and trimeric isoforms as they appear in human plasma, C) KRHBA buffer +20 µg/ml of pure trimeric adiponectin isoform (Adiponectin Human, Trimeric form, Biovendor, Brno, Czech Republic, D) KRHBA buffer +20 µg/ml of purified globular adiponectin representing 16.6 kDa C-terminal globular fraction of adiponectin (Recombinant Human gAcrp30/Adipolean, Peprotech, Inc., Princeton Business Park, USA) and E) KRHBA buffer +0.5 mM AICAR (pharmacological AMPK activator, aminoimidazole carboxamide ribonucleotide, Sigma-Aldrich, Prague, Czech Republic). Glycerol released into culture media was determined by colorimetric assay (Glycerol kit, Randox laboratories, Crumlin, United Kingdom) before and after incubations. Glycerol values were expressed per 100 mg of lipids determined by the Dole lipid extraction method [26] and used as a marker of lipolysis.

### Human Preadipocytes Differentiation

Human preadipocytes were derived from SCAAT biopsies obtained from an obese donor as described above. Cells were grown in 12-well collagen-coated plates at 5% CO_2_ and 37°C in DMEM/F12 Ham’s medium supplemented with 15 mM HEPES, 2.5 mM L-glutamine, 5% fetal calf serum and Preadipocyte Growth Medium Supplement Pack (Promocell, Heidelberg, Germany). Antibiotic Antimycotic Solution (Sigma-Aldrich, Prague, Czech Republic) was added to all media used. Preadipocytes were cultured until confluence and subsequently differentiated into adipocytes using DMEM/F12 Ham’s medium supplemented with 15 mM HEPES, 2.5 mM L-glutamine, 3% fetal calf serum, 33 µM biotin, 17 µM pantothenate, 1 µM dexamethasone, 0.2 nM isobutylmethylxanthine, 100 nM insulin and 10 µM rosiglitazone. Differentiation medium was removed after 3 days and cells were grown in identical medium but without rosiglitazone. Adipocytes were fully differentiated into mature adipocytes and used for experiments on the 10^th^ day after beginning of the differentiation protocol.

Preadipocytes used in experiments investigating adiponectin isoforms-induced AMPK activation (Thr172 phosphorylation) were obtained from a non-obese individual (Zen-Bio Inc., Research Triangle Park, NC) and differentiated according to manufacturer’s instructions using recommended differentiation and growth media (Zen-Bio Inc., Research Triangle Park, NC).

### Western Blot Analysis

Experiments were performed after 24-hour starvation in fresh serum-free DMEM/F12 Ham’s medium supplemented with 15 mM HEPES, 2.5 mM L-glutamine, 33 µM biotin and 17 µM pantothenate. Cells were pre-incubated for 1 hour in culture medium followed by 24 hours of treatment with pharmacological substances (i.e. AICAR, full-length, trimeric and globular adiponectin) in all experiments except those investigating Thr172 p-AMPK phosphorylation induced by adiponectin isoforms, where incubation time was 10 minutes. Control experiment was conducted without any pharmacological stimulation. After incubations, adipocytes were washed with ice-cold buffer composed of 20 mM Tris, 150 mM NaCl, 10 mM EDTA, 100 mM NaF, 10 mM pyrophosphate and 2 mM sodium orthovanadate, pH 7.4. Cells were lysed in identical buffer with 1% Triton X-100 and Phosphatase/protease inhibitors (Complete Protease Inhibitor Cocktail, Roche, Basel, Switzerland). Cell homogenates were centrifuged and infranatant used for protein quantification (BCA Assay, Thermo Fisher Scientific Inc., Waltham, MA, USA). After SDS-PAGE separation, proteins were transferred to a PVDF membrane, blocked with 5% bovine serum albumin (BSA)and incubated overnight with primary antibodies diluted 1∶1000 in 2% BSA in 0.1% TBS (0.1% Tween in Phosphate Buffered Saline). Following antibodies were used: 1) anti-phospho-Ser79-ACC rabbit polyclonal antibody, (Millipore, Billerica, MA, USA), 2) anti-phospho-Ser565-HSL rabbit polyclonal antibody, (Cell Signaling Technology, Beverly, MA, USA) and 3) anti-tubulin antibody (Cell Signaling Technology, Beverly, MA, USA), 4) PhosphoPlus® AMPKα (Thr172) Antibody Duet (Cell Signalling Technology, Inc., Danvers, MA, USA). Secondary antibody (HRP-coupled anti-rabbit secondary antibody, Jackson Immunoresearch Laboratories, West Grove, PA, USA) was diluted 1∶5000 in 5% milk in 0.1% TBS and membrane incubated for 1 hour at room temperature. Chemiluminescence was developed using the Pierce ECL Western Blotting Substrate (Thermo Fisher Scientific Inc., Rockford, IL, USA) and detected on Fujifilm Las-3000 apparatus (Fujifilm Life Science, Courbevoie, France) or Hyperfilm ECL (Amersham, Pittsburgh, PA, USA). Band intensities were digitalized and quantified using MultiGauge software (Fujifilm Europe GmbH, Dusseldorf, Germany) or ImageJ software and expressed as relative values normalized either to tubulin or total AMPK band intensity as appropriate.

### Adiponectin Receptors (AdipoR1 and AdipoR2) Gene Expression in SCAAT and VAT

Paired samples of SCAAT and VAT were obtained during planned laparoscopic surgery from a separate sample of obese (N = 9, age = 37.9±3.3 years, BMI = 34.5±1.4 kg/m^2^) and non-obese women (N = 6, age = 41.8±3.4 years, BMI = 21.1±3.7 kg/m^2^). Total RNA was extracted using the Trizol reagent (Life Technologies, Carlsbad, CA, USA). After assessment of the total RNA quality on an agarose gel, cDNA was synthesized using the iScript cDNA Synthesis Kit (BioRad Life Science Research, Hercules, CA, USA) and qPCR was performed with iCycler instrument (BioRad Life Science Research, Hercules, CA, USA) using the QuantiTect SYBR Green PCR Kit (Qiagen GmbH, Hilden, Germany). Expression levels of target genes were normalized to the geometric mean of expression levels of three housekeeping genes identified in our previous experiments and through literature search as most stable control genes in human adipose tissue: ACTB (β-actin), RPLP0 (60S acidic ribosomal protein P0) and PPIA (peptidylprolyl isomerase A). Specificity of primers and qPCR reaction was verified by DNA gels, sequencing of PCR products and melt curves. Reaction efficiencies for all genes were calculated from the standard curve (2-fold dilution) and used to calculate gene expression levels (Efficiency^-Ct^). Data are expressed as the ratio of the gene of interest expression/geometric mean of the expression of three control genes. Primers used in the qPCR are listed in Table 2.

**Table 2:** Primers used for qPCR analysis.

Gene	Primers
**ACTB**	F 5′-CCTTGCACATGCCGGAG- 3′
	R 5′-ACAGAGCCTCGCCTTTG- 3′
**RPLP0**	F 5′-TGTCTGCTCCCACAATGAAAC- 3′
	R 5′-TCGTCTTTAAACCCTGCGTG- 3′
**PPIA**	F 5′-TCTTTCACTTTGCCAAACACC- 3′
	R 5′-CATCCTAAAGCATACGGGTCC- 3′
**AdipoR1**	F 5′-AATTCCTGAGCGCTTCTTTCCT- 3
	R 5′-CATAGAAGTGGACAAAGGCTGC- 3′
**AdipoR2**	F 5′-ATGGCCAGCCTCTACATCAC- 3′
	R 5′-GCCGATCATGAAACGAAACT- 3

**Table 1:** Anthropometric and biochemical variables of subjects.

	Obese	Non-obese
**Age (years)**	48.1±3.9	54.7±5.3
**BMI (kg/m^2^)**	36.8±1.6	24.1±1.1
**Adiponectin (µg/mL)**	2.0±0.1	2.9±0.8
**Glycerol (µmol/L)**	111.2±11.9	68.1±25.8
**FFA (µmol/L)**	986.2±55.6	589.9±155.6*
**Insulin (mU/L)**	12.8±2.0	7.1±2.5
**Glucose (mmol/L)**	6.4±0.6	4.3±0.3
**Total cholesterol (mmol/L)**	4.9±0.3	4.5±1.1
**HDL-cholesterol (mmol/L)**	1.2±0.1	1.4±0.2
**Triglycerides (mmol/L)**	1.9±0.1	N/A

* p<0.05 for differences between obese and non-obese.

### Blood Analysis

Blood samples were obtained before surgery from an indwelling intravenous catheter and stored at −80°C until analysis. Plasma adiponectin was measured using DuoSet Human Adiponectin ELISA (R&D Systems, Inc., Minneapolis, USA). Plasma glycerol and FFA levels were determined by colorimetric assay (Glycerol kit and NEFA kit, Randox laboratories, Crumlin, United Kingdom). Plasma insulin concentration was determined using radio-immuno assay (Insulin IRMA KIT IM 3210, Beckman coulter-Immunotech, Czech republic). Plasma glucose, triglycerides, high-density lipoprotein and total cholesterol were determined photometrically using Glucose Hexokinase II, Cholesterol 2, Direct HDL Cholesterol and Triglycerides reagents (ADVIA 1800 Chemistry System Analyzer, Siemens Healthcare Diagnostics, Tarrytown, NY, USA).

### Statistical Analysis

Statistical analysis was performed using SPSS 13.0 for Windows (SPSS Inc., Chicago, IL,USA). The effect of pharmacological substances as well as differences between obese and non-obese groups were tested with non-parametric Wilcoxon’s test. Data are expressed as mean values ± SEM. A level of p≤0.05 was considered statistically significant in all tests.

## Results

### The Effect of AICAR and Adiponectin Isoforms on Spontaneous Lipolysis

**Figure 1:**
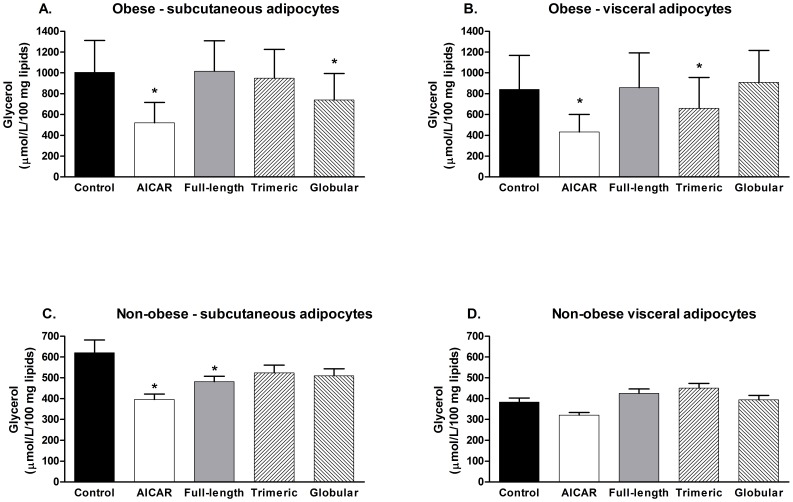
The effect of AICAR, full-length, trimeric and globular adiponectin isoforms on spontaneous lipolysis. Glycerol concentration in media after 2-hour incubation of adipocytes derived from (A) subcutaneous abdominal adipose tissue of obese subjects, (B) visceral/omental adipose tissue of obese subjects, (C) subcutaneous abdominal adipose tissue of non-obese subjects and (D) visceral/omental adipose tissue of non-obese subjects with 0.5 mM AICAR, 20 µg/ml full-length adiponectin, 20 µg/ml trimeric adiponectin and 20 µg/ml globular adiponectin * p<0.05 for comparison with the control experiment.

As summarized in Figure 1, in obese subjects globular isoform inhibited lipolysis in SCAAT (1004.9±292.1 versus 738.8±243.4 µmol/L/100 mg lipids, p<0.05) and trimeric isoform inhibited lipolysis in VAT (838.9±242.8 versus 660.3±218.8 µmol/L/100 mg lipids, p<0.05), while no effect of full-length adiponectin on lipolysis was observed. Treatment with AICAR suppressed lipolysis in both SCAAT and VAT by 48% and 49%, respectively (p<0.05) in obese subjects. In contrast, full length adiponectin and AICAR suppressed lipolysis in subcutaneous adipocytes of non-obese subjects by 22% and 36%, respectively (p<0.05), while the inhibitory effect of trimeric and globular isoforms remained non-significant (lipolysis inhibition by 15% and 18%, respectively). No effect of AICAR or any of the adiponectin isoforms was observed in VAT of non-obese individuals.

Spontaneous lipolysis in SCAAT but not VAT was positively associated with BMI (r = 0.56, p<0.05) in obese, while no association was observed in non-obese subjects in either localization. Basic anthropometric and biochemical characteristics of subjects enrolled in this study are summarized in Table1.

### The Effect of AICAR and Adiponectin Isoforms on AMPK, ACC and HSL Phosphorylation

**Figure 2:**
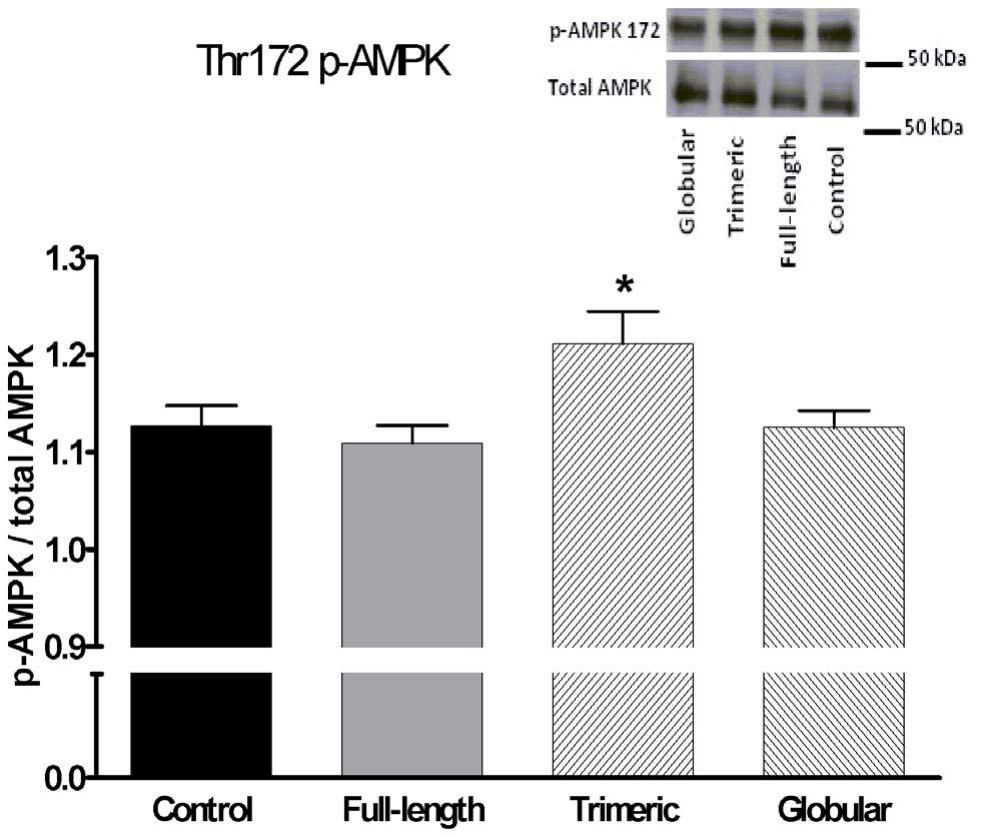
The effect of full-length, trimeric and globular adiponectin isoforms on Thr172 AMPK phosphorylation. (A) Levels of phospho-Thr172-AMPK were measured in differentiated preadipocytes derived from SCAAT of a non-obese donor after 10-minute treatment with 20 µg/ml full-length adiponectin, 20 µg/ml trimeric adiponectin and 20 µg/ml globular adiponectin. (B) A representative western blot of p-AMPK and total AMPK. Data are expressed as a ratio of p-AMPK/total AMPK. N = 5 for each treatment * p<0.05 for comparison with the control experiment.

**Figure 3:**
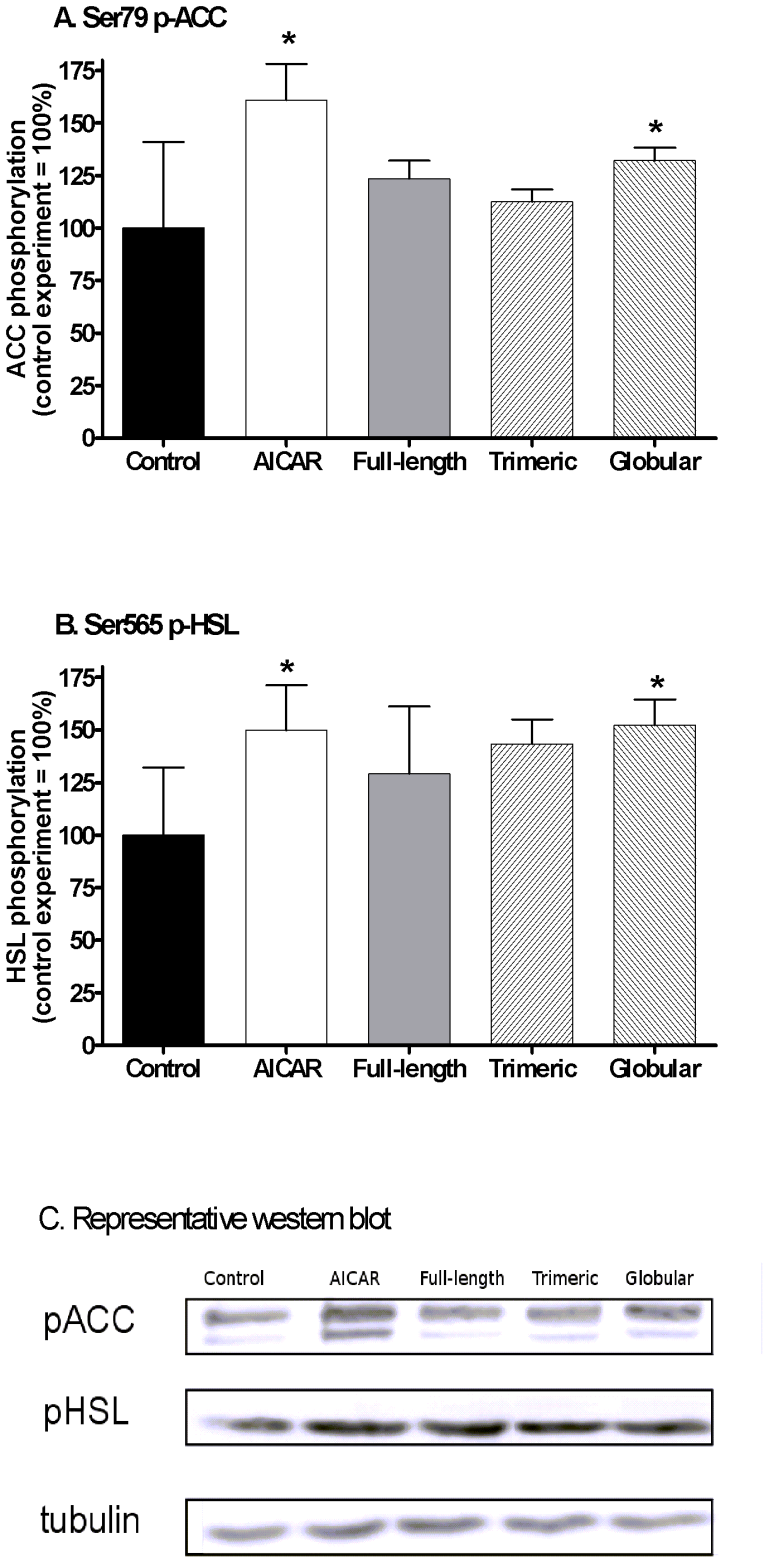
The effect of AICAR, full-length, trimeric and globular adiponectin isoforms on Ser79 ACC and Ser565 HSL phosphorylation. Levels of phospho-Ser79-ACC (A) and phospho-Ser565-HSL (B) were investigated in differentiated preadipocytes derived from SCAAT after 24-hour treatment with 2 mM AICAR, 10 µg/ml full-length adiponectin, 10 µg/ml trimeric adiponectin and 10 µg/ml globular adiponectin expressed as % change over the control experiment. (C) A representative western blot of p-ACC, p-HSL and tubulin as a loading control. N = 4 for each treatment * p<0.05 for comparison with the control experiment.

We studied the ability of adiponectin isoforms to acutely induce AMPK activation in differentiated human preadipocytes. In preadipocytes derived from a non-obese donor, we observed that only trimeric isoform induced detectable AMPK phosphorylation at Thr172 residue (Figure 2), while in preadipocytes derived from obese donors, globular isoform induced Ser79 p-ACC by 32% (p<0.05) and Ser565 p-HSL by 52% (p = 0.08) while full-length or trimeric isoforms had no effect (Figure 3). Treatment with AICAR increased Ser79 p-ACC by 60.9% (p<0.001) and Ser565 p-HSL by 50% (p = 0.09), while full-length or trimeric isoforms had no effect (Figure 2) in differentiated preadipocytes from obese donors.

### Gene Expression of Adiponectin Receptors in SCAAT and VAT of Obese and Non-obese Subjects

**Figure 4:**
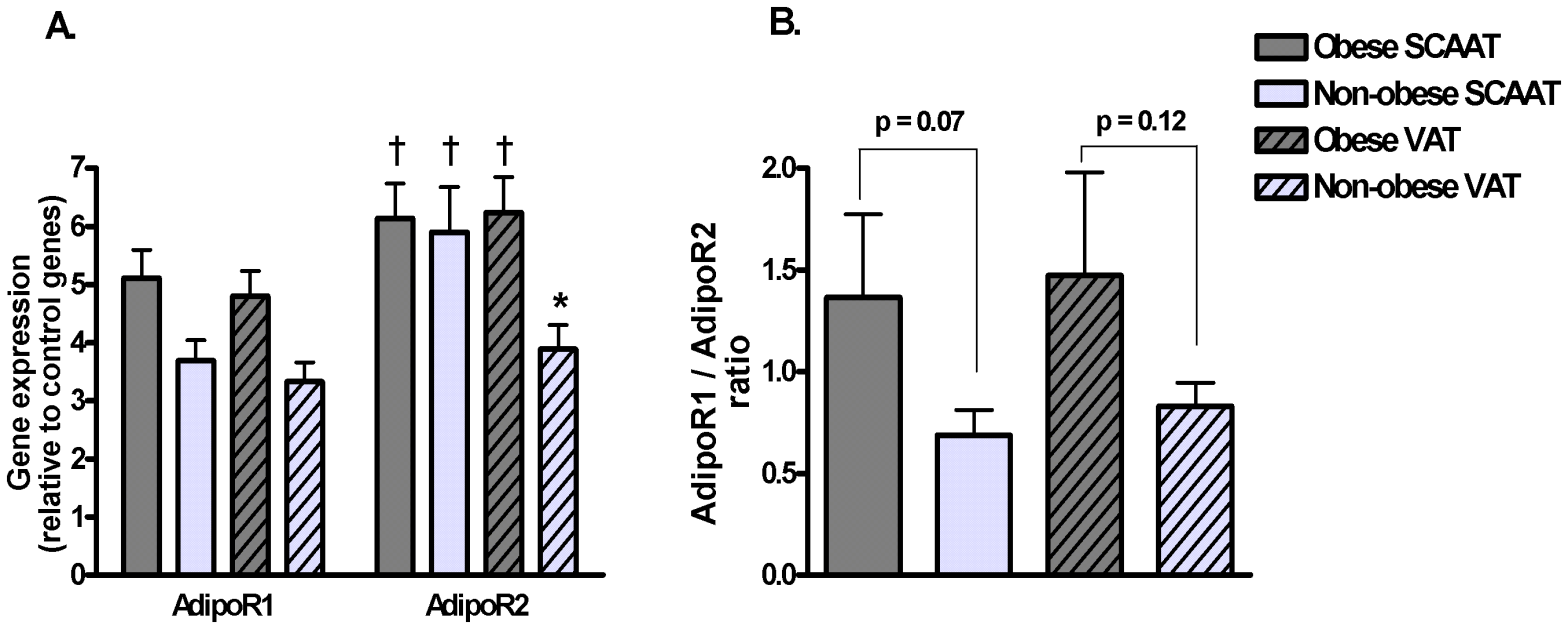
Gene expression of AdipoR1 and AdipoR2 in SCAAT and VAT of obese and non-obese subjects. (A) Relative gene expression of AdipoR1 and AdipoR2 in SCAAT and VAT of obese and non-obese subjects as the ratio of the gene of interest expression/geometric mean of the expression of three control genes (ACTB, RPLP0, PPIA). (B) Ratio of AdipoR1/AdipoR2 relative expression in SCAAT and VAT of both groups. N = 9 for obese and N = 6 for non-obese subjects * p<0.05 for comparison between SCAAT and VAT, ^†^ p<0.05 for comparison between AdipoR1 and AdipoR2 expression.

Expression of AdipoR2 was 17% and 37% higher than expression of AdipoR1 in SCAAT of obese and non-obese subjects, respectively (p<0.05). Similar finding was observed in VAT of obese individuals (23% higher expression of AdipoR2, p<0.05), while no differences in receptor expression was observed in VAT of non-obese individuals. As a result, obese subjects showed no depot differences in expression of both receptors, while non-obese obese subjects showed 34% higher expression of AdipoR2 in SCAAT compared to VAT (p<0.05). AdipoR1/AdipoR2 ratio was 49% and 43% higher in SCAAT and VAT of obese subjects as compared to non-obese group, however these results reached only borderline statistical significance (p = 0.07 and p = 0.12, respectively). Data are summarized in Figure 4.

## Discussion

The purpose of this study was to elucidate paracrine regulation of spontaneous lipolysis by full-length, trimeric and globular adiponectin isoforms in obese and non-obese subjects. Furthermore, depot-specific differences were investigated in subcutaneous and visceral adipose tissue. The major finding is that lipolysis is specifically inhibited by globular adiponectin in SCAAT and by trimeric isoform in VAT, while no effect of full-length adiponectin was observed in either depot of obese subjects, while full-length adiponectin was effective in SCAAT of non-obese subjects. We also showed that in differentiated human preadipocytes obtained from an obese donor, only globular adiponectin induced ACC and HSL phosphorylation at amino-acid residues specific for AMPK activity, while in differentiated adipocytes obtained from a non-obese donor, trimeric adiponectin most effectively induced AMPK activation.

Adiponectin is secreted in adipose tissue predominantly in a multimeric form [27] and subsequently circulates in plasma as multimeric, hexameric, trimeric and globular isoforms [13], [17]. Although the biological significance of individual isoforms is not sufficiently understood, it has been shown that several metabolic effects are dependent on adiponectin polymerization state. For example, full-length adiponectin induced AMPK activation both in muscle and liver [21], while trimeric and globular isoforms were effective only in muscle [13], [17], [23], [28]. Differential biological effects of individual adiponectin isoforms are further demonstrated by studies showing that globular isoform, but not full-length adiponectin, induced specific intracellular signaling, prevented cell adhesion and reduced hyperglycemia induced damage in endothelial cells [29], [30]. Indeed, even mutually opposite effects of globular and full-length adiponectin on reactive oxygen species production in phagocytes were reported [31]. In the present study, we observed that adiponectin-induced inhibition of lipolysis is also dependent on its polymerization state and obesity status. While in non-obese individuals full-length adiponectin suppresses lipolysis in SCATT, as we have shown here as well as in a previous study [14], this effects is lost with the development of obesity. In contrast, SCAAT adipocytes of obese individuals demonstrated anti-lipolytic sensitivity to the globular fragment of adiponectin. Similar modifications in adiponectin sensitivity were observed in the VAT, where no effect of either isoform was observed in non-obese individuals, but trimeric isoform suppressed lipolysis in obese subjects. Important differences in lipolysis regulation between SCAAT and VAT were described previously [14], [20], [32], however, we provided evidence in this study that also adiponectin-mediated lipolysis regulation is modified by adipose tissue localization and modified with obesity.

Mechanisms mediating adiponectin isoform-specificity and depot-specificity have not been clarified so far. Tissue-specific expression of adiponectin receptors might play a role as both types of adiponectin receptors are expressed in adipocytes [33]. Importantly, globular and trimeric isoforms bind preferentially to AdipoR1 receptor (adiponectin receptor type 1), while multimeric isoforms bind AdipoR1 and AdipoR2 (adiponectin receptor type 2) more equally [34]. We hypothesize, that relative distribution of AdipoR1 and AdipoR2 receptors in adipose tissue determines biological response to individual adiponectin isoforms. Interestingly, obese subjects showed higher AdipoR1/AdipoR2 ratio (representing relatively lower abundance of AdipoR2) in subcutaneous as well as visceral adipose tissues when compared to non-obese group. Although expression of AdipoR1 and AdipoR2 was not different between SCAAT and VAT in obese subjects, as previously reported [35], [36], distribution of both receptors in VAT varied between obese and non-obese subjects in our study. It can be speculated, that limited quantity of AdipoR2, preferentially activated by higher-molecular weight adiponectin isoforms, might explain the lack of effect of the full-length adiponectin in obese subjects as well as the preserved activity of the globular isoform, activating strongly AdipoR1 [34].

Metabolic effects of adiponectin are mediated through activation of multiple intracellular signaling cascades including PPARα, MAPK, PKA and AMPK pathways [34], [37]. We were particularly interested in AMPK pathway because several metabolic processes in the cell including lipolysis are regulated by this master energy switch [22], [24], [34]. AMPK-induced phosphorylation of HSL at Ser565 prevents subsequent phosphorylation of HSL at the regulatory Ser563 position [7] necessary for HSL-induced lipolytic activity. Pharmacological activation of AMPK by AICAR in our study efficiently suppressed lipolysis in all fat depots except non-obese VAT, documenting preserved functionality of signaling mechanisms downstream of AMPK and suggesting that either modified receptor affinity to individual adiponectin isoforms or intracellular signaling events upstream of AMPK mediate the depot- and isoform-specificity of adiponectin isoforms. We have documented the ability of the trimeric isoform to acutely (10 minutes) induce AMPK phosphorylation at its regulatory site (Thr172) in differentiated preadipocytes derived from SCAAT of a non-obese donor, however, only globular adiponectin induced phosphorylation of Ser79 p-ACC and Ser565 p-HSL (amino acid residues specific for AMPK activity [24], [38]) after prolonged treatment (24 hours) in differentiated adipocytes derived from SCAAT of an obese donor. These results provide additional support for the conclusion that obesity reduces sensitivity to higher molecular weight adiponectin isoforms with globular adiponectin exerting anti-lipolytic activity in SCAAT. Such a conclusion is congruent with adiponectin receptor distribution data described above. Due to the limited availability and quantity of VAT, we were unable to test this hypothesis in VAT and it thus remains unclear whether identical regulations are present in VAT.

Depot and isoform specificity of adiponectin isoforms might also be regulated by tissue factors determining local adiponectin isoform availability. For example, quantity of globular adiponectin in tissues is locally regulated by the activity of monocyte-derived elastase releasing globular fraction from higher molecular form isoforms [39]. It has also been suggested that multimeric adiponectin might compete with other isoforms for receptor binding [22]. Whether these factors are differentially regulated in SCAAT versus VAT and/or obese versus non-obese subjects remains to be determined.

Although AMPK activation by adiponectin was observed in rat adipocytes [7], [20] (only globular isoform was studied), AMPK-independent mechanisms of adiponectin-induced anti-lipolytic effects were recently reported in mouse adipose tissue [37], where adiponectin increased degradation of protein kinase A regulatory subunit - limiting thus the phosphorylation and activation of HSL [37]. This finding is in agreement with the notion that adiponectin exerts its action by signaling mechanisms including activation of cAMP/PKA as well as AMPK pathways [40], [41]. Furthermore, mutual interactions between PKA and AMPK pathways was reported [40], [42], [43], however precise mediators of such interactions remain unknown. Further research needs to address whether adiponectin isoforms differentially induce activation of PKA and/or AMPK in adipocytes and what are the functional consequences.

The present study directly compared effects of three adiponectin isoforms using concentrations ranging in the upper-end of plasma physiological concentrations; however our *ex-vivo* study has clear limitations. First, experiments in isolated adipocytes do not fully reflect the physiological situation *in-vivo,* where complex interactions exist between adipocytes, other cell types in adipose tissue and multiple hormones. Second, adiponectin concentrations used in our incubations were ∼2–3 times higher than plasma levels typically observed in obese individuals, however, interstitial adipose tissue concentration (determining paracrine effects) of adiponectin as well as other adipokines are typically significantly higher compared plasma levels [7], [44]. It should also be noted, that adipocytes release *de-novo* synthesized adiponectin into the culture media. Based on our previous studies, we calculated that the secreted adiponectin represented ∼12–15% of the exogenous adiponectin in media at the end of incubations [45]. Third, it should be noted that the full-length adiponectin used in our study represents a mixture multimeric, hexameric and trimeric isoform. Purified recombinant multimeric or hexameric isoform is not commercially available and we were not successful in chromatography-based isolation of these isoforms in a sufficient purity suitable for *in-vitro* studies. Finally, it should be noted that conclusions on the lack of effect of various polymeric isoforms throughout this paper should be evaluated with caution due to the possibility of type II. statistical error. Future studies focusing on the role of adiponectin in stimulated lipolysis regulation (e.g. during exercise or prolonged fasting) are warranted.

In conclusion, we documented that lipolysis inhibition induced by adiponectin is modified with the development of obesity and different between SCAAT and VAT. While full-length adiponectin has an anti-lipolytic effect in SCAAT and no effect in VAT of non-obese individuals, these regulations are changed in obesity, where globular isoform is active in SCATT and trimeric isoform in VAT. We suggest that these differences reflect the isoform-specific ability to activate AMPK in adipocytes and the distribution of AdipoR1 and AdipoR2 receptors which is modified by obesity. We hypothesize that in obesity lower molecular weight adiponectin isoforms (globular and trimeric) become important factors regulating plasma FFA levels through the suppression of adipose tissue lipolysis and increased fatty acid utilization in muscle [17], [23], [28]. As elevated plasma FFA levels were causally linked to the pathogenesis of insulin resistance and β-cell dysfunction, adiponectin-induced inhibition of lipolysis represents additional mechanism for anti-diabetic effects of adiponectin [3], [4], [46]. Supposedly, modifying the quantity of adiponectin isoforms by pharmacological compounds would provide a future tool for the lipotoxicity prevention and treatment in obese subjects.
